# The PHD transcription factor Cti6 is involved in the fungal colonization and aflatoxin B1 biological synthesis of *Aspergillus flavus*

**DOI:** 10.1186/s43008-021-00062-2

**Published:** 2021-05-18

**Authors:** Zhang Mengjuan, Lin Guanglan, Pan Xiaohua, Song Weitao, Tan Can, Chen Xuan, Yang Yanling, Zhuang Zhenhong

**Affiliations:** Key Laboratory of Pathogenic Fungi and Mycotoxins of Fujian Province, Key Laboratory of Biopesticide and Chemical Biology of Education Ministry, Proteomic Research Center, and School of Life Sciences, Fujian Agriculture and Forestry University, Fuzhou, 350002 China

**Keywords:** *Aspergillus flavus*, Cti6, PHD domain, AFB1, Colonization

## Abstract

*Aspergillus flavus* and its main secondary metabolite AFB1 pose a serious threat to several important crops worldwide. Recently, it has been reported that some PHD family transcription factors are involved in the morphogenesis and AFB1 biological synthesis in *A. flavus*, but the role of Cti6, a PHD domain containing protein in *A. flavus*, is totally unknown. The study was designed to reveal the biological function of Cti6 in the fungus by deletion of *cti6*, and its two domains (PHD and Atrophin-1) through homologous recombination, respectively. The results showed that Cti6 might up-regulate the mycelium growth, conidiation, sclerotia formation and AFB1 biological synthesis of *A. flavus* by its PHD domain, while Atrophin-1 also improved the conidiation of the fungus. The qRT-PCR analysis showed that Cti6 increased the conidiation of the fungus through AbaA and BrlA mediated conidiation pathway, triggered the formation of sclerotia by orthodox sclerotia formation pathway, and improved the production of AFB1 by orthodox AFB1 synthesis pathway. Crops models analysis showed that *A. flavus* Cti6 plays vital role in colonization and the production of AFB1 on the host grains mainly via PHD domain. Bioinformatics analysis showed Cti6 is conservative in *Aspergillus spp.,* and mCherry mediated subcellular localization showed that most Cti6 accumulated in the nuclei, which reflected that Cti6 performed its important biological function in the nuclei in *Aspergillus spp..* The results of the current study elucidate the roles of PHD domain containing proteins in the mechanism of the infection of crops by *A. flavus,* and provided a novel target for effectively controlling the contamination of *Aspergillus spp.* to crops.

**Supplementary Information:**

The online version contains supplementary material available at 10.1186/s43008-021-00062-2.

## INTRODUCTION

As a soil saprophyte worldwide, the notorious *Aspergillus flavus* colonizes many important crops, such as corn, peanut and cotton, and the threat of the pathogen to the life of immunosuppressed patients through aspergillosis is just second to *A. fumigatus* (Amaike and Keller 2011; Hedayati et al. 2007; Tsui et al. 2011). This pathogenic fungus also causes aflatoxicosis to animal and human through the contamination of crop and feed by its most toxic secondary metabolites among known mycotoxins: aflatoxins (including aflatoxin B1, B2, G1 and G2) (Amaike and Keller 2011; Tumukunde et al. 2020). Among aflatoxins, aflatoxin B1 (AFB1) is known to be the most toxic and carcinogenic mycotoxins, and it is one of the Group 1 carcinogens listed by IARC (the International Agency for Research on Cancer) (Wu et al. 2014; Xing et al. 2016). It is critical to reduce the detriment of *Aspergillus flavus* to crop, animal and human by manipulating the regulating mechanism of reproduction or secondary metabolite formation in such a way to stop/suppress the SM/reproduction.

The morphogenesis, reproduction and secondary metabolism of *A. flavus* are regulated by several global transcriptional regulatory factors. As one of the global regulators, VeA is necessary for the production of mycotoxins, including AFB1, B2, cyclopianic acid, aflatrem, and sclerotia in *A. flavus* (Duran et al. 2007). The nuclear regulator LaeA is found to regulate the secondary metabolism in both *A. nidulans* and *A. fumigatus*, while in *A. flavus*, the global regulator LaeA negatively regulates of VeA, and is involved in the production conidia, sclerotia and aflatoxin (Kale et al. 2008).

The first PHD (plant homeodomain) finger domain was found in HAT3.1 protein of Arabidopsis (Schindler et al. 1993). Several PHD finger proteins have been found in various eukaryotic species, mainly in plants and animals, while the biological function of most of them are still unidentified (Aasland et al. 1995; Wang et al. 2015). The morphotype (yeast or hypha form) of *Cryptococcus neoformans* is tightly linked to the virulence of this pathogenic fungus, and 5 PHD finger domain containing genes among the genome of this pathogen were found to profoundly affect the filamentation of this pathogen (Meng et al. 2018). Our previous study revealed that the PHD family transcription factors are involved in the morphogenesis and aflatoxin biological synthesis in *A. flavus,* which showed that the PHD family transcription factor Rum1 represses conidiation, increases sclerotia formation and aflatoxin biological synthesis, up-regulates amylase activity of *A. flavus*, and involves in the colonization of the pathogenic fungus on crop kernels (Hu et al. 2018). By blast with the sequence of the main functional PHD domain of Rum1, Cti6 was found to be a PHD domain containing protein in *A. flavus*. In *Saccharomyces cerevisiae*, Cti6 was reported to be involved in the growth of yeast cells under iron-limiting condition (Puig et al. 2004). In addition, Cti6 was found to act in concert with SAGA (Spt-Ada-Gcn5-acetyltranferase) to alleviate Cyc8-Tup1 mediated repression and improve transcriptional activation in yeast (Papamichos-Chronakis et al. 2002). However the biological function of the PHD transcription factor Cti6 in *A. flavus* has not been explored. The current study was conducted to reveal the functions of Cti6 in the virulence of *A. flavus*, and to find a new target for the early control of the contamination of the pathogenic fungus.

## MATERIALS AND METHODS

### Strains and media

*A. flavus* Δ*ku70*Δ*pyrG* was used as the original strain in this work. All the strains used in this study are listed in Table 1. *A. flavus* was cultured in plates containing potato dextrose agar (PDA, 39 g/L, BD Difco, Franklin, NJ, USA), CM (6 g/L tryptone, 6 g/L yeast extract, 10 g/L glucose), or YES media (20 g/L yeast extract, 150 g/L sucrose, 1 g/L MgSO_4_•7H_2_O). For solid media, agar was added at 15 g/L. The auxotrophic marker (*pyrG*-) was supplemented with uracil and uridine each at 1 mg/mL in media.

**Table 1:** *A. flavus* strains in the study

Fungal strains	Genotype description	Reference
*A. flavus* CA14	Δ*pyrG,* Δ*ku70*	Purchased from FGSC
Control (Ctrl)	Δ*ku70,* Δ*pyrG::pyrG*	Used in our lab
Δ*cti6*	Δ*ku70,* Δ*cti6::pyrG*	This study
Com-*cti6*	Δ*ku70,* Δ*cti6::pyrG, cti6::pyrG*	This study
*cti6*^ΔPHD^	Δ*ku70,* Δ*pyrG,* Δ*PHD:pyrG*	This study
*cti6*^ΔATR^	Δ*ku70,* Δ*pyrG,* Δ*ATR:pyrG*	This study
*mcherry-cti6*	Δ*ku70,* Δ*pyrG, mcherry-cti6:pyrG*	This study

### Preparation of mutant strains

The *cti6* gene deletion strains were prepared according to the method of homologous recombination. 5′- flanking region (with primer p1 and p2, primers used in strain construction were listed in Table S1) and 3′ - flanking region (primer p3 and p4) of cti6 were amplified, and fused together with *A. fumigatus pyrG* by nesting primer p7 and p8 according to the strategy scheme shown in Figure S1A. The fusion PCR product was transformed into the *A. flavus* CA14 strain by polyethylene glycol-mediated approach (Zhuang et al. 2016). Transformants were selected on a medium without Uracil and Uridine, and confirmed with PCR, RT-PCR, qRT-PCR and southern-blotting analysis. For the complementation assay, the *pyrG* in Δ*cti6* was replaced by 5′-flanking region-*cti6*–3′-flanking region (amplified with primer p1 and p4) under the stress of 2 mg/mL 5-FOA (5-fluorooroticacid) with the method of homologous recombination. Then, *pyrG* gene was inserted into the transformants at the N-terminal of *cti6* gene by homologous recombination with the fusion production amplified by cti6-C-p1 and cti6-C-p4 primers as shown in the strategy scheme in Figure S1B. Finally, the constructed Com-cti6 strain was further verified using diagnostic PCR, RT-PCR and qRT-PCR. For functional domain deletion mutants, *cti6*^ΔPHD^ and *cti6*^ΔATR^, the construction principle was same as the construction of Δ*cti6* strain, and the mutant strains were confirmed by PCR and DNA sequencing in BioSune Biotechnology (Shanghai, China).

### Bioinformatics analysis

The homologs of Cti6 (from *A. flavus*, *A. oryzae*, *A. terreus*, *A. fumigatus*, *A. nidulans*, *Pyricularia oryzae*, *S. cerevisiae*, *Homo sapiens*, *Mus musculus* and *Arabidopsis thaliana*) were downloaded from NCBI (https://pubmed.ncbi.nlm.nih.gov/), and their evolutionary relationship was analyzed with MEGA5.0 (Kumar et al. 2016). The domains of Cti6 were further identified through NCBI database (XP_002383836.1) and visualized by DOG2.0^2^.

### qRT-PCR analysis

Fungal spores (10^6^/mL) were coating culture on PDA medium (for conidiation), CM medium (for sclerotia formation) or YES (for AFB1 production) for 48 h. Thereafter, mycelium was ground into powder with liquid nitrogen, and each 50 mg mycelium powder was lysis in 1 mL Trizol reagent (Biomarker Technologies, Beijing, China) for 5 min. Then the total RNA was prepared according to previous protocol with minor modification (Yang et al. 2019). The total RNA was reversely transcribed into cDNA using First Strand cDNA Synthesis Kit with oligo (dt) 18 primer (TransGen Biotech, Beijing, China). The qRT-PCR was performed following the protocol formerly described (Hu et al. 2018), and the primers used in qRT-PCR were shown in Table S2.

### Phenotype analysis

For mycelium growth analysis, spores (10^3^) was point-inoculated on 15 mL PDA in petri dish at 37 °C in dark, and the diameter of each fungal colony was measured after 5 d. To count the conidia number, 4 cores (10 mm in diameter) along the radius of the colony were drilled, after which these cores were immersed in 3 mL water in a 6 mL Falcon tube. The spore suspension was transferred into a new Eppendorf tube, after the Falcon tube was vortexed, and the spores was counted under the microscope (Hu et al. 2018). For sclerotia formation analysis, the spores (10^3^) were point-cultured on CM medium at 37 °C in dark for 7 d, and the sclerotia number was counted when the fungal colony was sprayed with 70% ethanol (Hu et al. 2018).

### Analysis of AFB1 production

The AFB1 production analysis was performed according to the method previously described (Hu et al. 2018). The fungal spores (10^6^/mL) of each *A. flavus* strain were inoculated into 10 mL of YES liquid medium, and cultured in the dark at 29 °C for 6 d. Afterwards, AFB1 was extracted by mixing 2 mL YES liquid medium from the fungal culture with an equal volume of methylene chloride. The extracted AFB1 was further analyzed with TLC (thin layer chromatography) with silica gel plate (Macklin, shanghai). The AFB1 production of different *A. flavus* strain was assessed by relative quantitative analysis against the AFB1 standard sample (0.1 mg/mL).

### Subcellular localization

The construction of *mcherry-cti6* strain was done according to previously described protocol (Liu et al. 2020). The fungal spores (10^4^) were inoculated in YES liquid medium at 37 °C for 12 h. For this purpose, the mycelia were collected by centrifugation at a speed of 13,000 rpm for 20 min. And the collected mycelium was rinsed with PBS, then, was observed after staining with DAPI (Bioss, Beijing) for 10 min by laser confocal scanning microscope (LeicaSP8).

### Iron concentration stress test

In the study carried out to examine the role of Cti6 in the growth of *A. flavus* under iron stress condition, the liquid iron-stress medium was prepared according to a previous report on *A. fumigatus* with minor modifications (Reiber et al. 2005). The fungal strains Ctrl, Δ*cti6, cti6*^ΔPHD^, *cti6*^ΔATR^ and Com-*cti6* were inoculated in the liquid iron-stress medium (25 g/L glucose, 3.5 g/L (NH_4_)_2_SO_4_, 2.0 g/L KH_2_PO_4_, 0.5 g/L MgSO_4_ and 8 mg/L ZnSO_4_, pH 6.8), and supplied it with serial concentration of Fe (III)Cl_3_ (0, 5, 10, 15 and 20 μM) as required. The liquid iron-stress medium with 20 μM Fe (III)Cl_3_ was set as the control medium without iron stress. After 7 d in 37 °C culture, the wet and dry mycelia were weighed, and the relative inhibition rates of iron stress on mycelia growth were assessed (The relative inhibition rate = (the weight of mycelium from the medium with 20 μM Fe (III)Cl_3_ - the weight of mycelium from each iron stress medium with from 0 to 15 μM Fe (III)Cl_3_)/the weight of mycelium from the medium with 20 μM Fe (III)Cl_3_).

### Crop colonization assays

The peanut and corn infection models were established based on previously described protocol (Hu et al. 2018). After aseptically treated with 8% sodium hypochlorite, peanuts and corn grains were soaked with 10^5^/mL fungal spores of each *A. flavus* strain for 30 min. The kernels were incubated in the dark at 29 °C for 6 days, then, photographic observation was carried out. AFB1 was extracted by soaking the crop kernels in 5 mL dichloromethane for 20 min. The production levels of AFB1 in different fungal strain treated groups were analyzed by TLC.

### Statistical analysis

All data in this study were presented with the means ± standard deviation. The analysis of statistics was implemented with the software GraphPad Prism5 (La Jolla, CA, USA) using the Tow-way ANOVA of Tukey test analysis, and the difference was regarded to be statistically significant when *p* < 0.05. Error bars represented standard error for at least three replicates.

## RESULTS

### Cti6 is conservative in *Aspergillus spp*.

**Fig. 1:**
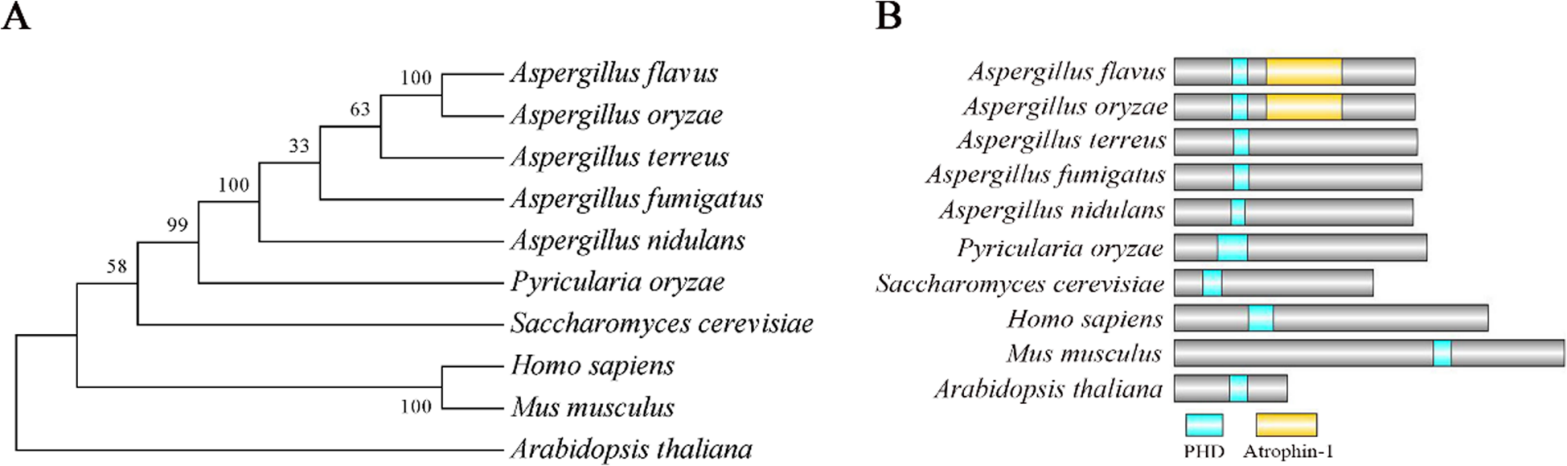
Bioinformatics analysis of Cti6. **a** The construction of phylogenetic relationship among 10 Cti6 homologs (from *A. flavus*, *A. oryzae*, *A. terreus*, *A. fumigatus*, *A. nidulans*, *P. oryzae*, *S. cerevisiae*, *H. sapiens*, *M. musculus* and *A. thaliana*) with MEGA5.0. **b** The domains of Cti6 from above 10 species were identified through NCBI, and the domains were further visualized by DOG2.0^2^

The evolutionary relationship of 10 Set1 homologs (from *A. flavus*, *A. oryzae*, *A. terreus*, *A. fumigatus*, *A. nidulans*, *Pyricularia oryzae*, *S. cerevisiae*, *Homo sapiens*, *Mus musculus* and *Arabidopsis thaliana*) was analyzed with MEGA5.0. It showed that the Cti6 homologs from the fungal species were classified into one group, in which the highest similarity (100% Identity, 100% Query Cover) was identified between *A. flavus* and *A. oryzae*, whereas the lowest similarity (48.21% Identity, 9% Query Cover) was found between *A. flavus* and *S. cerevisiae* (Fig. 1**a**). Cti6 is more conserved among *Aspergillus spp.*, the lowest similarity is between *A. flavus* and *A. nidulans* (68.55% Identity, 100% Query Cover) in the cladogram. The domains in Cti6 were further identified through NCBI database (Accession No.: XP_002383836.1), and visualized by DOG2.0^2^. A PHD (plant homeodomain) and a Atrophin-1 domain were found from the Cti6 homologs of both *A. flavus* and *A. oryzae*, and only PHD domain could be found in all 10 homologous proteins (Fig. 1b).

### Cti6 is involved in mycelium growth and conidiation of *A. flavus*

To evaluate the biological function of Cti6 in the growth, development and virulence of *A. flavus*, *cit6* gene deletion strain (Δ*cti6*) and its complementary strain (Com-*cti6*) were constructed according to the strategy of homologous recombination as shown in Figure S1A and S1B. The constructed Δ*cti6* and Com-*cti6 A. flavus* strains were further validated with diagnostic PCR, RT-PCR, qRT-PCR and southern-blotting, the results showed that both Δ*cti6* and Com-*cti6* strains are successfully constructed (Figure S1C to S1F). To assess the role of the PHD and the Atrophin-1 domain inside Cti6, both domains were deleted with the method of homologous recombination Figure S2A and S2C, and the resulted PHD deletion strain (*cti6*^ΔPHD^) and Atrophin-1 deletion strain (*cti6*^ΔATR^) was further confirmed by sequencing as shown in Figure S2B and S2D.

**Fig. 2:**
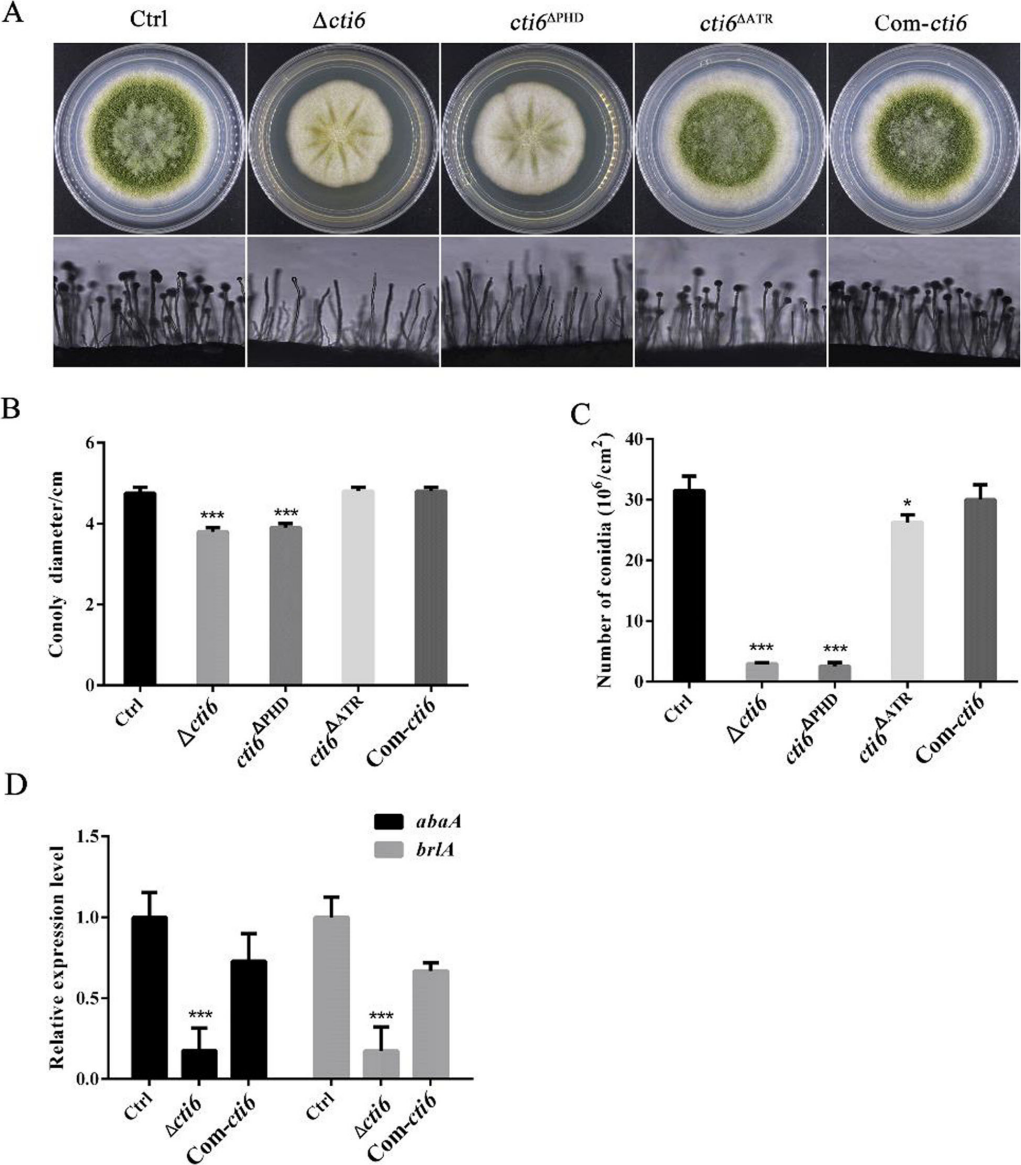
Cti6 up-regulates the growth of fungal mycelium and conidiation in *A. flavus*. **a** The *A. flavus* strains were point-inoculated on PDA media for 5 d at 37 °C. **b** The colony diameters were measured and represented with column graph according to the result of (**a**) panel. **c** The number of conidia of each fungal strain on PDA medium. **d** The expression level of transcriptional factor *abaA* and *brlA* genes. The “*”, “**” and “***” represents significant difference levels: *p* < 0.01, *p* < 0.005 and *p* < 0.001, respectively. All experiments were carried out with three biological replicates, and repeated at least three times

To explore the biological function of Cti6 and its two domains in the growth and conidiation of *A. flavus*, the constructed fungal strains were point-inoculated on PDA media for 5 d. It showed that the mycelium became white and fluffy, and the colony became smaller than that of Ctrl strain when Cti6 was absent (Fig. 2a upper panel). From the microscopic observation, few conidia could be found on the top of sporophore in the Δ*cti6* mutant (Fig. 2a lower panel). To domain deletion strains, the results showed that the conidiation state and the size of the colony of *cti6*^ΔPHD^ were similar to that of the Δ*cti6,* but Atrophin-1 domain exhibited an insignificant effect on the mycelium growth of *A. flavus* (Fig. 2a). The column graph established according to the size of fungal colonies reflected that Cti6 and the PHD domain in Cti6 played a significant role in the growth of *A. flavus* (Fig. 2b). The conidia number was counted with hemocytometer, and the resulted column graph showed that the absence of Cti6 (*p* < 0.001), PHD domain (*p* < 0.001) or Atrophin-1 (*p* < 0.01) domain significantly reduced the conidiation capacity of the fungus (Fig. 2c). Further qRT-PCR analysis, as shown in Fig. 2d, indicated that Cti6 up-regulated the conidiation of *A. flavus* through AbaA (*p* < 0.005) and BrlA (*p* < 0.001). The results suggested that Cti6 regulated conidiation through AbaA and BrlA mediated conidiation pathway, and PHD domain played a critical role in the process.

### Cti6 is indispensable for the sclerotia formation in *A. flavus*

**Fig. 3:**
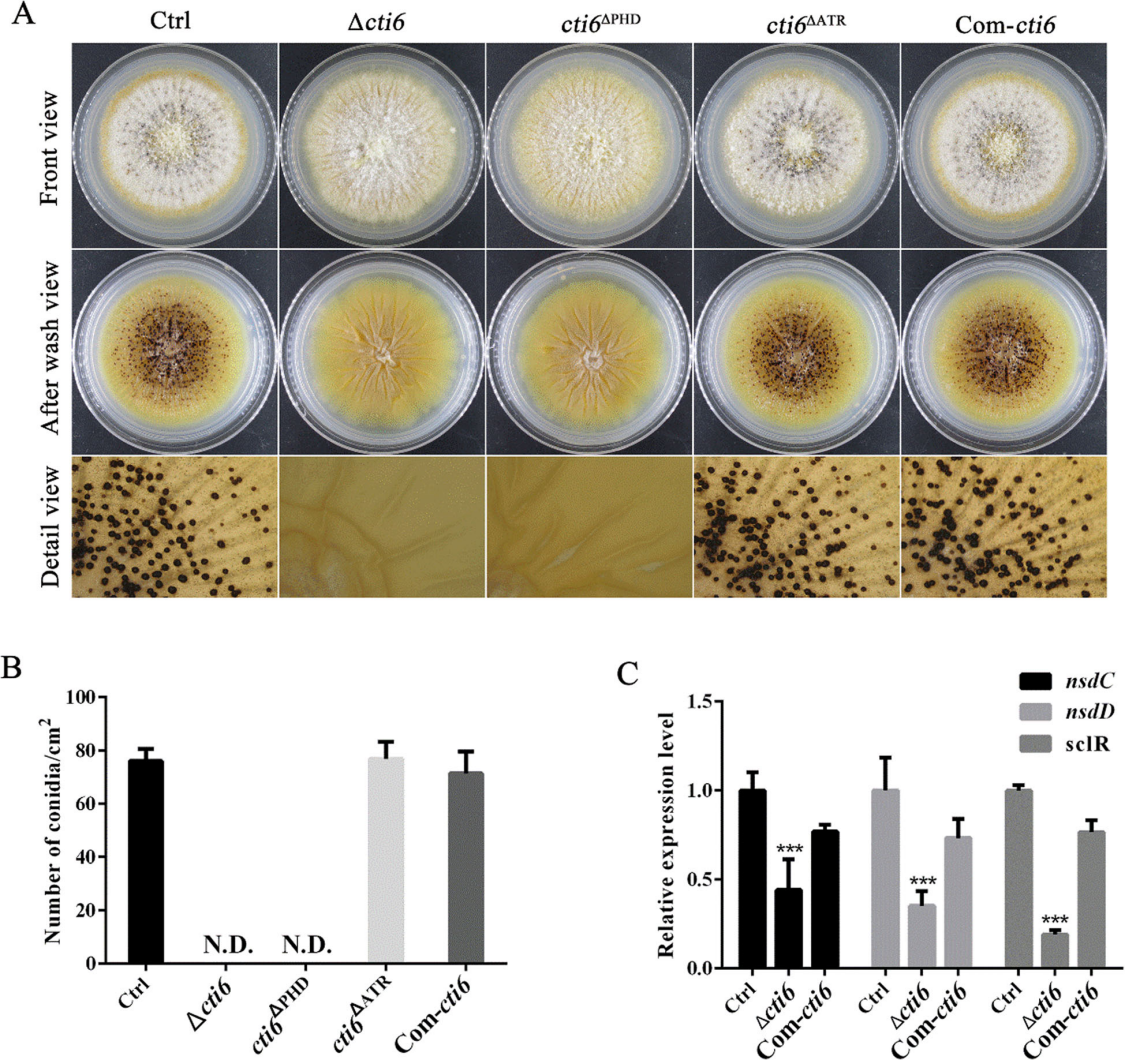
Cti6 is necessary in the formation of sclerotia in *A. flavus*. **a** The *A. flavus* strains were point-inoculated on CM media for 7 d at 37 °C. **b** The sclerotia number was counted, and the sclerotia formation ability of these fungal strains was compared. **c** The expression levels of transcriptional factor genes (*nsdC, nsdD* and *sclR*) were analyzed by qRT-PCR

To evaluate the role of Cti6 in the formation of sclerotia in *A. flavus,* the constructed fungal strains were point-inoculated on CM media for 7 d. The results showed that no sclerotia was formed when Cti6 or the PHD domain inside Cti6 was missing (Fig. 3a). The sclerotia number was calculated, and the resulted Fig. 3b showed that the deletion of *cti6* gene (Δ*cti6* strain) or deletion of the PHD domain from the gene (*cti6*^ΔPHD^ strain) total inhibit the formation of sclerotium, and the absence of Atrophin-1 domain had no effect in the formation of sclerotia compared to Ctrl. The expression levels of *nsdC, nsdD* and *sclR* were further monitored by qRT-PCR, and the result showed that the deletion of *cti6* gene significantly decreased the expression level of these three key sclerotia formation regulators. Above results showed that Cti6 regulated the formation of sclerotia by orthodox sclerotia formation pathway.

### Cti6 is critical for the biological synthesis of AFB1 in *A. flavus*

**Fig. 4:**
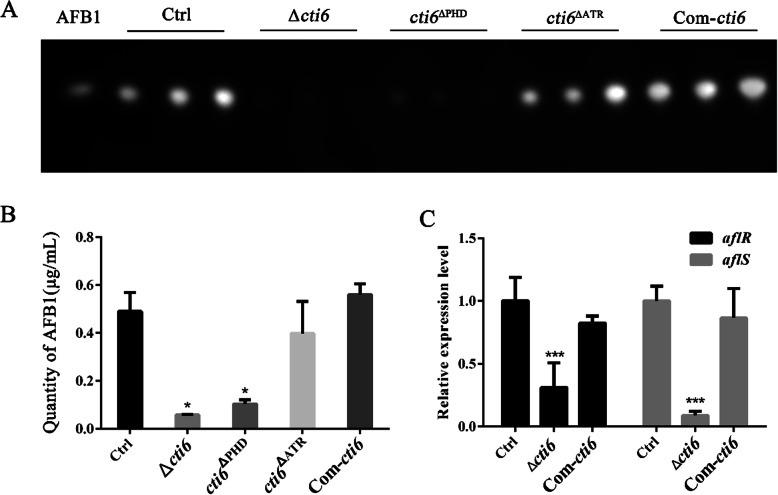
Cti6 is critical in AFB1 synthesis in *A. flavus*. **a** The production of AFB1 from above fungal strains was analyzed with TLC. **b** The relative quantity of the amount of AFB1 according to the result from above TLC analysis. **c** The expression levels of AFB1 synthesis regulator gene *aflR* and *aflS* were analyzed by qRT-PCR

In the study conducted to evaluate the biological functions of Cti6 in AFB1 synthesis, the fungal strains (Ctrl, Δ*cti6, cti6*^ΔPHD^, *cti6*^ΔATR^ and Com-*cti6*) were cultured with liquid YES at 29 °C for 6 d. Aflatoxins were extracted by dichloromethane, and analyzed by TLC. As shown in Fig. 4a and b, when Cti6 or the PHD domain inside Cti6 was absent, the production of AFB1 was dramatically decreased. On the other hand, Atrophin-1 domain appears not to participate in the process. Further qRT-PCR analysis revealed that the regulator genes *aflR* and *aflS* in the orthodox AFB1 synthesis pathway were significantly down-regulated when Cti6 was absent (Fig. 4c). The aforementioned results suggested that Cti6 regulated the biological synthesis of AFB1 via its PHD domain through AflR regulated AFB1 synthesis pathway.

### Cti6 is involved in the colonization of *A. flavus* to crops

**Fig. 5:**
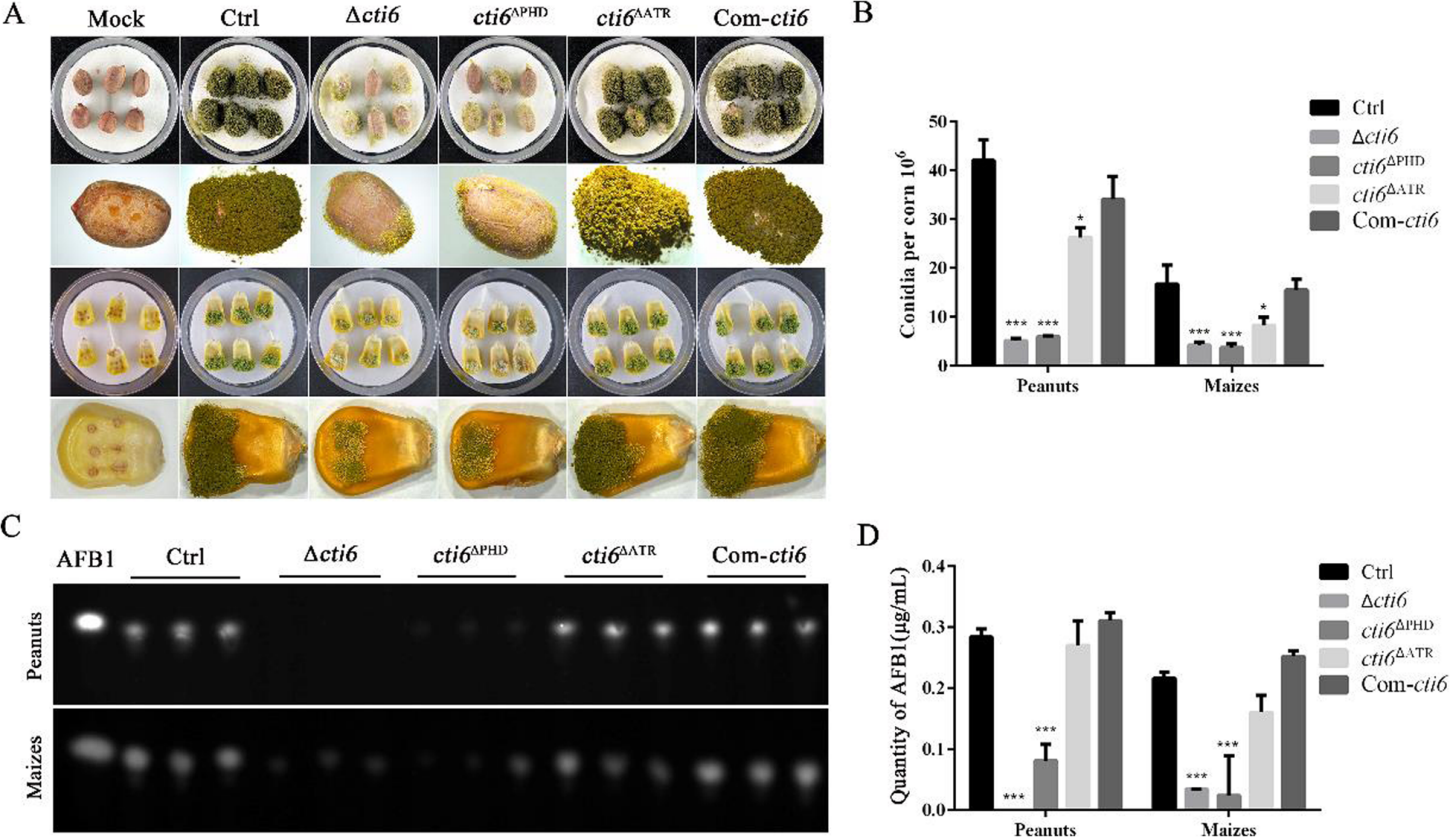
The role of Cti6 in the colonization of *A. flavus* on peanut and maize grains. **a** Colonization of these *A. flavus* strains on peanut and maize grains. **b** Cti6 and its PHD domain involved in the conidiation of *A. flavus*. **c** The AFB1 production capacity of these fungal strains was analyzed with TLC analysis. **d** Cti6 and its PHD domain positively regulated AFB1 production in *A. flavus*

*A. flavus* contaminates various kinds of crop kernels, especially oil plant, including peanuts and maize. To assess the role of Cti6 and its main domains in the colonization of *A. flavus* on crop grains, we inoculated the plant kernels (peanut and maize) with the spores from Ctrl, Δ*cti6, cti6*^ΔPHD^, *cti6*^ΔATR^ and Com-*cti6* according to the protocol mentioned in the Materials and Methods. The results revealed that the colonization ability of Δ*cti6* and *cti6*^ΔPHD^ strains on peanut and maize grains decreased dramatically compared to Ctrl and Com-*cti6* strains (Fig. 5a), and the sporulation capacity of the fungi without Cti6 (*p* < 0.001) or its domains PHD (*p* < 0.001) and Atrophin-1 (*p* < 0.01) was inhibited significantly compared to the Ctrl (Fig. 5b) in both crop models (Fig. 5a). The main mycotoxin of *A. flavus* - AFB1 was further extracted from the fungus contaminated kernels using methylene chloride, and analyzed with TLC (Fig. 5c). The results from TLC showed that the AFB1 mycotoxin producing capacity of both Δ*cti6* and *cti6*^ΔPHD^ decreased drastically compared to the Ctrl and any other fungal strains used in this study in both crop grain models (Fig. 5c). Further statistical analysis revealed that the absence of Cti6 and PHD domain dramatically reduced the production of AFB1 (*p* < 0.001, Fig. 5d). Above results hinted that Cti6 of *A. flavus* plays critical role in colonization and AFB1 production on host crop grains mainly via PHD domain, but Arophin-1 domain in the *cti6* gene doesn’t involved in these biological processes.

### Localization of Cti6 in the nucleus of *A. flavus*

**Fig. 6:**
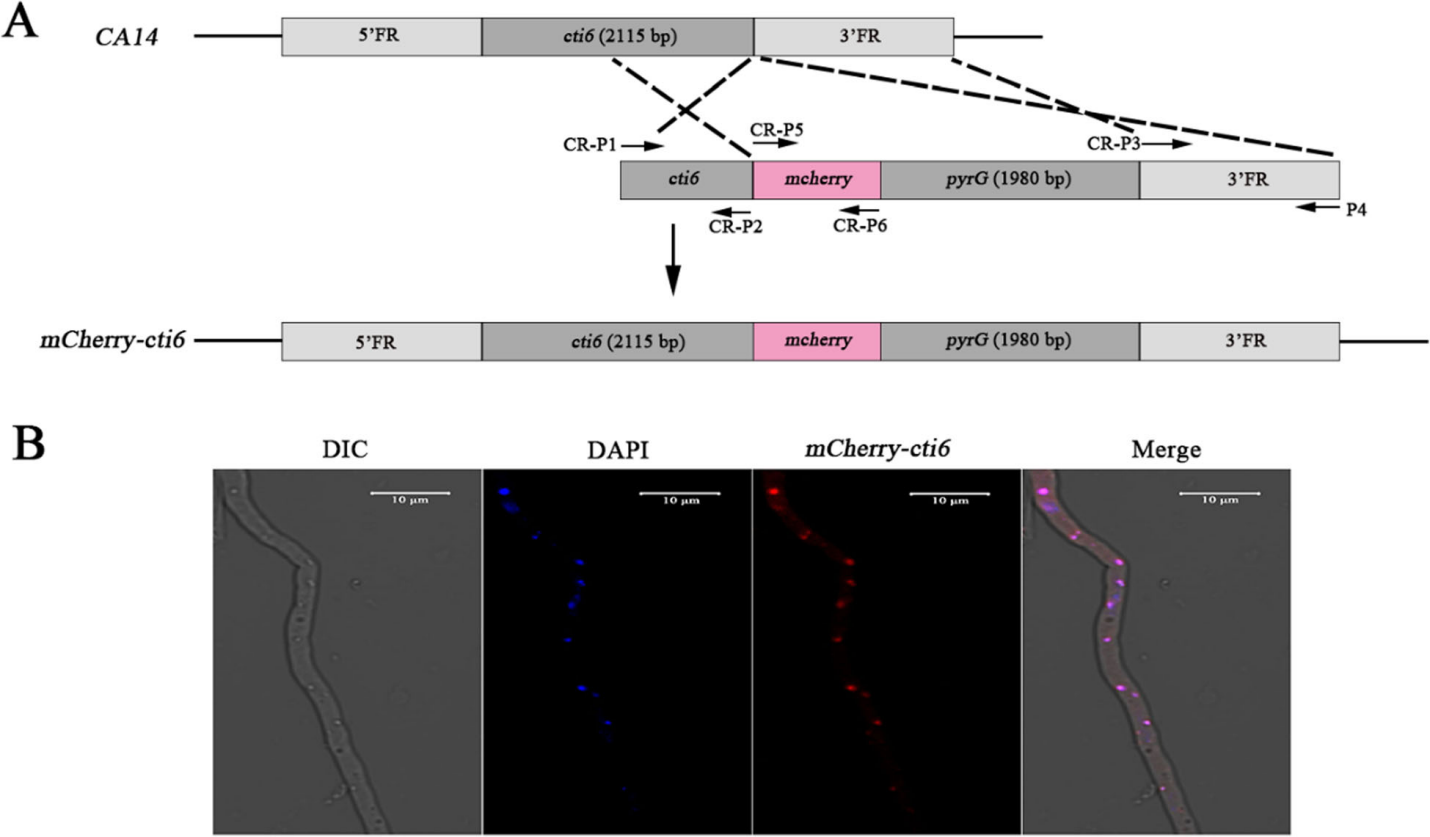
The subcelluar location of Cti6. **a** The construction strategy for *mCherry* and *cti6* fusion expression *A. flavus* strain (*mCherry-cti6*). **b** The subcellular location of Cti6 was showed through co-expressed mCherry

To examine the subcellular localization of Cti6 in *A. flavus*, a fungal strain in which Cti6 was tagged with mCherry at its N-terminus was constructed with the strategy of homologous recombination as shown in Fig. 6a. The subcellular position of Cti6-mCherry was localized with a 552 nm light source by the laser confocal scanning microscope (LeicaSP8), and the position of nuclei was identified with a light source of 405 nm wavelength. By dual-channel imaging, Cti6 was found to be accumulated in the nuclei (Fig. 6b).

## DISCUSSION

### The PHD transcription factor Cti6 is conservative in *Aspergillus spp.*, and accumulated in the nuclei of *A. flavus*

Phylogenetic analysis showed that the Cti6 homologs from *A. flavus*, *A. oryzae*, *A. terreus*, *A. fumigatus* and *A. nidulans* are clustered into the same group, and the lowest similarity of Cti6 homologs among *Aspergillus spp.* in the cladogram is 68.55% (between *A. flavus* and *A. nidulans,* Fig. 1a). The Cti6 homologs from *A. flavus* and *A. oryzae* are identical (100% Identity, 100% Query Cover), and both of them harbor a PHD and a Atrophin-1 domain, while Atrophin-1 domain could not be found in any other Cti6 homologs among these 10 species (Fig. 1b). This outcome suggested that the PHD zinc fingers domain is more conservative and more important in proper execution of the biological function of Cti6 than Atrophin-1 domain. Atrophin-1 is reported to be responsible for DRPLA (dentatorubral-pallidoluysian atrophy), a progressive neurodegenerative disorder (Wood et al. 1998). PHD domain has been reported to be involved in chromatin mediated transcriptional regulation by reading covalently modification histone sequence (Aasland et al. 1995; Arrowsmith and Schapira 2019; Sanchez and Zhou 2011). By co-location with mCherry, Cti6 was found to be accumulated in the nuclei of the fungus (Fig. 6b). The results suggested that the PHD transcriptional factor Cti6 is conservative in *Aspergillus spp*, and implements similar important biological functions in the nuclei of these filamentous fungi. However, there is no available report on the biological function of Cti6 in the filamentous fungi until now.

### Cti6 improves mycelium growth and asexual development in *A. flavus*

By point-inoculated on the PDA media or inoculated on the grains of corn and peanut, it was found that Cti6 positively improves the growth of the fungal mycelium, and up-regulates conidiation of *A. flavus* by transcriptional factor AbaA and BrlA (Figs. 2 and 5). BrlA and AbaA are two key DNA-binding transcriptional factors in regulation of asexual proliferation, in which BrlA initiates the program, and AbaA activates the development of the phialides in conidiophores (the asexual fruiting bodies) of filamentous fungi (Mead et al. 2020; Sewall 1994). By deletion of PHD and Atrophin-1 domain, it was found that, similar to Δ*cti6*, the fungal colony size, mycelial density and conidiation state of PHD domain deletion strain (*cti6*^ΔPHD^) on both PDA media and crop kernels were significantly restrained compared to the Ctrl fungal strain, but Atrophin-1 domain was only involved in the sporulation of the fungus (Figs. 2 and 5). The results of the study inferred that Cti6 regulates mycelium growth and asexual reproduction mainly via the assistance of PHD domain, and the PHD transcriptional factor improves the conidiation of *A. flavus* through AbaA and BrlA mediated sporulation-specific pathway. In view of the fact that pathogenic filamentous fungi mainly contaminate crops through their spores, the results of the current study showed Cti6 and its PHD domain are ideal potential targets for reducing the colonization rate of these pathogens on various kinds of important crops.

### Cti6 is required in the formation of sclerotia in *A. flavus*

The analysis on the role of Cti6 in the formation of sclerotia in the study showed that Cti6 is indispensable for the formation of the structure in *A. flavus* (Fig. 3a and b). As melanized hyphal aggregates, sclerotia are commonly considered a kind of alternative reproduction form and survival structures against adverse conditions in filamentous fungi (Chang et al. 2012). It was also reported in 2009 that ascocarps, the structures of *A. flavus* sexual state, was embedded inside sclerotia (Horn et al. 2009).

The helix-loop-helix transcriptional factor SclR*,* the GATA-type transcriptional factor *nsdD* and the C2H2-Type Transcriptional Factor *nsdC* are necessary for sexual development of filamentous fungi (Han et al. 2001; Jin et al. 2011; Kim et al. 2009). The results of this study reflected that Cti6 is required in the sexual reproduction of *A. flavus* by orthodox sclerotia regulatory transcriptional factors (NsdC, NsdD and SclR) regulated orthodox sclerotia formation pathway (Fig. 3c). By the construction of PHD and Atrophin-1 domain deletion mutants, it was found that no sclerotia was formed when PHD domain was deleted, which reflected that the PHD domain plays a key role in regulating the formation of sclerotia by Cti6 (Fig. 3). In *Aspergillus spp.* some regulators are very critical for the formation of sclerotia. To *A. parasticus*, sclerotia is also a resistant structure for survival under adverse conditions, and the deletion of the global regulatory factor *veA* lead to the blockage of sclerotia formation (Calvo et al. 2004). Therefore, Cti6 and its key domain PHD are very important breakthrough points to greatly reduce the genetic variation and survival rate of pathogenic filamentous fungi in unfavorable environmental conditions (such as in the periods of extreme cold or drought) by elimination of the structure of sclerotia.

### Cti6 plays a key role in biological synthesis of AFB1 in *A. flavus*

As the most toxic natural compound, AFB1, mainly produced by *A. flavus*, causes significant losses to agriculture. The role of Cti6 in biological synthesis of AFB1 in *A. flavus* was analyzed in the study. Following inoculation in liquid YES or on the kernels of corn and peanut, it was found that the absence of Cti6 greatly decreased the production of AFB1 in *A. flavus* by regulating the expression level of the aflatoxin biological synthesis regulatory genes *aflR* and *aflS* (Figs. 4a, c and 5c). With a GAL4-type binuclear zinc finger motif, the aflatoxin biological synthesis transcription factor AflR controls the biological synthesis of aflatoxin via regulating gene expression in the aflatoxin gene cluster (Liu and Chu 1998; Masanga et al. 2015; Woloshuk et al. 1994). AflR interacts with aflatoxin biological synthesis regulator AflJ via Arg427, Arg 429 and Arg 431 in its C-terminal region, and the deletion of AflJ significantly decreases the expression of genes *pksA*, *nor1*, *ver1* and *omtA* in the aflatoxin biological synthesis pathway, which results in the block the synthesis of AFB1 (Chang 2003). The deletion of Atrophin-1 domain from Cti6 obviously did not affect the production of AFB1 either in YES media or on the crop grains (Figs. 4a and 5c). However, the absence of PHD domain significantly reduced the biological synthesis level of AFB1 both in YES liquid and on crop kernels. This outcome was similar to what was recorded in the Δ*cti6* strain (Figs. 4a and 5c). These findings revealed that Cti6 plays an important role in the biological synthesis of aflatoxins mainly via its PHD domain through regulating the regulators AflR and AflJ, and that Cti6 and its PHD domain are good targets to reduce the aflatoxin contamination of crops that were polluted by *A. flavus*.

### Cti6 doesn’t involve in the growth of hyphae under iron stress

Cti6 was reported to play a role in the growth of yeast under iron-limiting condition (Puig et al. 2004). To check the role of Cti6 in *A. flavus* under iron stress, the relative inhibition rates of the fungal strains Ctrl, Δ*cti6, cti6*^ΔPHD^, *cti6*^ΔATR^ and Com-*cti6* inoculated in series liquid iron-stress medium was examined. The results of the iron stress test by relative inhibition rates analysis showed that Cti6 isn’t involved in the growth of *A. flavus* under iron stress conditions (Figure S3). In addition, comprehensive consideration based on the aforementioned results and the results from the bioinformatics analyses reflected that the biological functions of Cti6 might be conservative just among *Aspergillus spp.*.

In conclusion, this study revealed that Cti6 and its PHD domain play very important roles in the morphogenesis, mycotoxin biosynthesis, and crop colonization of *A. flavus* (Figure S4), and provided ideal targets to reduce the contamination rate of *A. flavus* on crops, and to reduce aflatoxin pollution of harvested crops. In general, our study provides insight into the potential biological functions of PHD domain containing transcriptional factors in the pathogenicity of filamentous fungi.

## Supplementary Information

**Additional file 1: Table S1.** Primers used in A. flavus strain construction. **Table S2.** Primers used in qRT-PCR. **Figure S1.** The construction of Cti6 deletion and complementary strains. **Figure S2.** The construction of PHD domain and Atrophin-1 domain deletion *A. flavus* strains. **Figure S3.** The iron stress effects in *A. flavus* strains. **Figure S4.** The regulation model showing the pathways by which Cti6 regulates the morphogenesis and aflatoxin biological synthesis, and colonization of *A. flavus*.

## Data Availability

All data generated or analysed during this study are included in this published article. The raw data supporting the conclusions of this article will be made available by the authors, without undue reservation, to any qualified researcher.

## Abbreviations

PHD domain: plant homeodomain finger superfamily
AFB1: aflatoxin B1
SM: secondary metabolism
TLC: thin layer chromatography
