# Supplementary material for: The PHD transcription factor Cti6 is involved in the fungal colonization and aflatoxin B1 biological synthesis of Aspergillus flavus
Source: IMA Fungus. 2021 May 18;12:12. doi: 10.1186/s43008-021-00062-2 (PMC8130384; doi:10.1186/s43008-021-00062-2)
Supplement: Supplementary file 1 — Additional file 1: Table S1. Primers used in A. flavus strain construction. Table S2. Primers used in qRT-PCR. Figure S1. The construction of Cti6 deletion and complementary strains. Figure S2. The construction of PHD domain and Atrophin-1 domain deletion A. flavus strains. Figure S3. The iron stress effects in A. flavus strains. Figure S4. The regulation model showing the pathways by which Cti6 regulates the morphogenesis and aflatoxin biological synthesis, and colonization of A. flavus. [file 43008_2021_62_MOESM1_ESM.docx]

**Supplementary Tables**

**Table S1 Primers used in *A. flavus* strain construction.**

| Primer Name | Sequence（5’-3’） | Fragment |
| --- | --- | --- |
| p1 | ATCACTCCTTGTTATCTCAACTCTCGCATCG | 5’FR of *cti6* |
| p2 | GGGTGAAGAGCATTGTTTGAGGC GGGCGGCGTGTATATAAGGTGGGA |  |
| p3 | GCATCAGTGCCTCCTCTCAGAC  CAGCATCATTCTTGCTTTGGGTTCCTCTCTC | 3’FR of *cti6* |
| p4 | CTTCCGTATTCCTTGCCCTGTTGTAGTTATGC |  |
| p5 | GCCTCAAACAATGCTCTTCACCC | A. fumigatus *pyrG* |
| p6 | GTCTGAGAGGAGGCACTGATGC |  |
| p7 | CTCCCACGAAGATTGACTTATTGCGAGGTACAC | Nesting primers |
| p8 | GATCACCGCGACCTGTAGGTATAGATGCAC |  |
| p9 | ATGACTCCTCGTCGCTCCTCTCGT | A fragment in *cti6* |
| p10 | TCACCTCTCGCCGAATTTCCCATACTCTTG |  |
| P801 | CAGGAGTTCTCGGGTTGTCG | *pyrG* testing primers |
| P1020 | ATCGGCAATACCGTCCAGAAGC |  |
| C-p1 | CAGCATCATTCTTGCTTTGGGTTCCTCTCTC | 5’FR of Com-*cti6* |
| C-p2 | GGGTGAAGAGCATTGTTTGAGGC  GATCACCGCGACCTGTAGGTATAGATGCAC |  |
| C-p3 | GCATCAGTGCCTCCTCTCAGAC  GCATAACTACAACAGGGCAAGGAATACGGAAG | 3’FR of Com-*cti6* |
| C-p4 | TCATACCCTCGCTTCCAAGAGACTCCCAAG |  |
| C-p7 | CGGCTACTGGAGCAACACTTTAAACTTGGAC | Nesting primers of Com-*cti6* |
| C-p8 | CGTCTATAACACCAACGCTAGCTATCAGCAGTC |  |
| CR-P1 | CAATCCTAAGCGTCGGTCGACGATGAAC | 5’FR of *mCherry*-*cti6* |
| CR-P2 | CCTCGCCCTTGCTCACCAT  CCTCTCGCCGAATTTCCCATACTCTTGC |  |
| CR-P3 | GCATCAGTGCCTCCTCTCAGAC  CGGCTACTGGAGCAACACTTTAAACT | 3’FR of *mCherry*-*cti6* |
| P4 | CTTCCGTATTCCTTGCCCTGTTGTAGTTATGC |  |
| CR-P5 | ATGGTGAGCAAGGGCGAG | *mCherry* |
| CR-P6 | GGGTGAAGAGCATTGTTTGAGGC CTTGTACAGCTCGTCCAT |  |
| CR-P7 | GGTATCCCAGCCCGCATCAGAAGAC | Nesting primers of *mCherry*-*cti6* |
| P8 | GATCACCGCGACCTGTAGGTATAGATGCAC |  |
| D1-1F | GGAAGAAACGGGAGGGAAGGAATGTAAAGTCG | 5’FR amplification in *cti6*^ΔPHD^ |
| D1-1R | CATTAAGTCCGACGAGTCTGCAGAGAAGTTCA |  |
| D1-2F | TGAACTTCTCTGCAGACTCGTCGGACTTAATG CGCAAAGATCTTCACAGAATTAAGAACGAGTCA | Primes for PHD deletion in *cti6*^ΔPHD^ strain |
| D1-2R | gggtgaagagcattgtttgaggc GAGAGAGGAACCCAAAGCAAGAATGATGCTG |  |
| D1-3F | gcatcagtgcctcctctcagac CGGCTACTGGAGCAACACTTTAAACTTGGAC | 3’FR amplification in *cti6*^ΔPHD^ |
| p4 | CTTCCGTATTCCTTGCCCTGTTGTAGTTATGC |  |
| D1-NF | CCTGGGTTAGAGGGGAAATTTTGGTGGAATGG | Nesting primers for *cti6*^ΔPHD^ |
| p8 | GATCACCGCGACCTGTAGGTATAGATGCAC |  |
| D1-NF | CCTGGGTTAGAGGGGAAATTTTGGTGGAATGG | 5’FR amplification in *cti6*^ΔATR^ |
| D2-1R | AGAATCTTTCGTCCGCTTAGAATTATCGCGGGA |  |
| D2-2F | TCCCGCGATAATTCTAAGCGGACGAAAGATTCT CCCGCACGCCGTGGTGG | Primes for ATR deletion in *cti6*^ΔATR^ |
| D1-2R | gggtgaagagcattgtttgaggc GAGAGAGGAACCCAAAGCAAGAATGATGCTG |  |
| D1-3F | gcatcagtgcctcctctcagac CGGCTACTGGAGCAACACTTTAAACTTGGAC | 3’FR amplification in *cti6*^ΔATR^ |
| p4 | CTTCCGTATTCCTTGCCCTGTTGTAGTTATGC |  |
| D2-NF | TCCCACCTTATATACACGCCGCCCT | Nesting primers for *cti6*^ΔATR^ |
| p8 | GATCACCGCGACCTGTAGGTATAGATGCAC |  |
| D1-TF | TCGCCCTTTGCCGACTCACTAT | Sequence primers of *cti6*^ΔPHD^ |
| D1-TR | CTCAATGGCGTCTCCAAGGAAC |  |
| D2-TF | GGGGTAAGGCTTTGATGTAGGG | Sequence primers of *cti6*^ΔATR^ |
| D2-TR | AGTGTTGCTCCAGTAGCCGTAAT |  |

**Table S2. Primers used in qRT-PCR.**

| Gene | Forward Sequences(5’-3’) | Reverse Sequences(5’-3’) |
| --- | --- | --- |
| *cit6* | GATGTCAATGGCAATGGAGACTCAAGCTTCATG | CACATATCGGGGTCGGCTAGGTTTCCCTG |
| *brlA* | GCCTCCAGCGTCAACCTTC | TCTCTTCAAATGCTCTTGCCTC |
| *abaA* | TCTTCGGTTGATGGATGATTTC | CCGTTGGGAGGCTGGGT |
| *nsdC* | GCCAGACTTGCCAATCAC | CATCCACCTTGCCCTTTA |
| *nsdD* | GGACTTGCGGGTCGTGCTA | AGAACGCTGGGTCTGGTGC |
| *sclR* | CAATGAGCCTATGGGAGTGG | ATCTTCGCCCGAGTGGTT |
| *aflR* | AAAGCACCCTGTCTTCCCTAAC | GAAGAGGTGGGTCAGTGTTTGTAG |
| *aflS* | CGAGTCGCTCAGGCGCTCAA | GCTCAGACTGACCGCCGCTC |
| *actin* | ACGGTGTCGTCACAAACTGG | CGGTTGGACTTAGGGTTGATAG |

**Supplementary Figures**

**
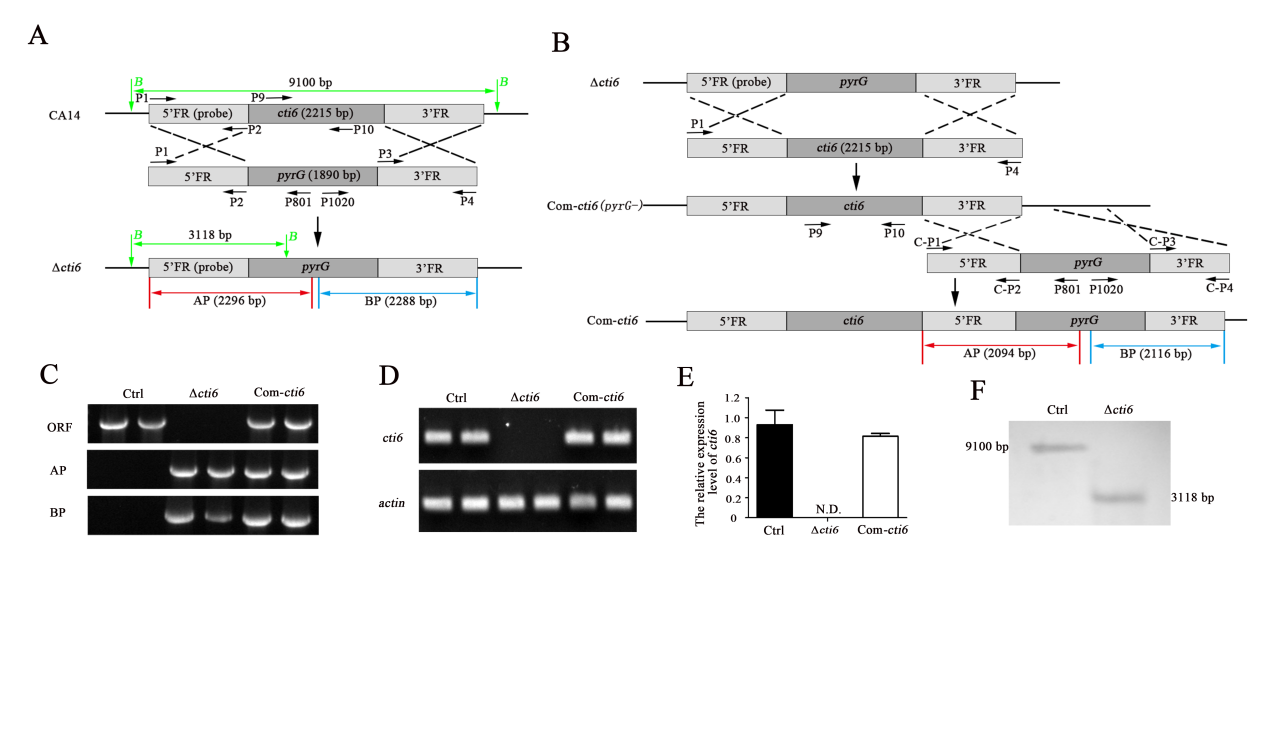
**

**Figure S1. The construction of Cti6 deletion and complementary strains. A.** The strategy for the construction of c*ti6* gene deletion *A. flavus* strain (Δ*cti6*). **B.** The construction strategy of c*ti6* gene complementary strain (Com-*cti6*). FR stands for the flanking region. **C.** The constructed Δ*cti6* and Com-*cti6* strains were validated by diagnostic PCR with genomic DNA as template. DNA fragment AP was amplifified with primer p1 and p801, BP with primer p1020 and p4, and ORF with primer p9 and p10 as shown in the strategy panel (A), and Ctrl is for control *A. flavus* strain. **D.** The *cti6* ORF in the Ctrl, Δ*cti6* and Com-*cti6* strains was tested by RT-PCR using cDNA as template, and *actin* was used as inner reference (the primers were listed in **Table S2**). **E.** The expression level of *cti6* in above *A. flavus* strains was monitored with qRT-PCR. **F.** *cti6* gene deletion mutant (Δ*cti6*) was identified by southern-blotting analysis. Genomic DNA from Ctrl and Δ*cti6* strains were digested by *BamH1* (*B*) and hybridized with a 1.49 kb probe (the 5’- flanking region of *cti6*), and the probe was amplifified with primers p1 and p2 as listed in **Table S1**.

**
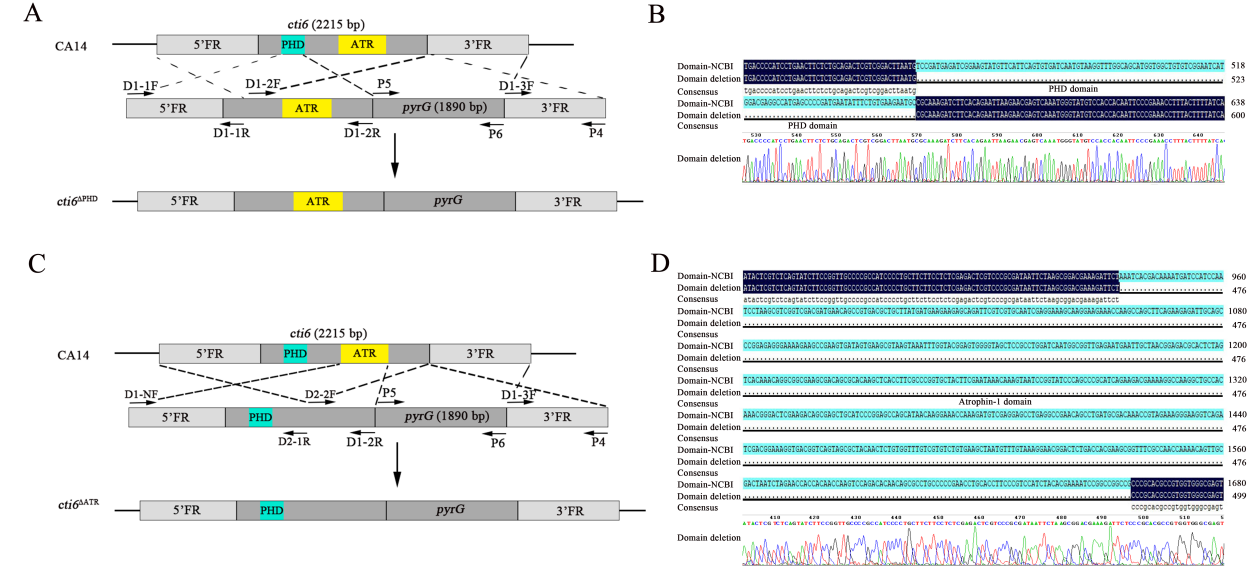
**

**Figure S2.** The construction of PHD domain and Atrophin-1 domain deletion *A. flavus* strains. **A.** The PHD domain deletion *A. flavus* strain (*cti6*^ΔPHD^) was constructed by homologous recombination. **B.** The resulted *cti6*^ΔPHD^ fungal strain was confirmed by sequencing. **C.** The strategy for the construction of Atrophin-1 domain deletion strain (*cti6*^ΔATR^). **D.** The constructed *cti6*^ΔATR^ *A. flavus* strain was tested by sequencing.


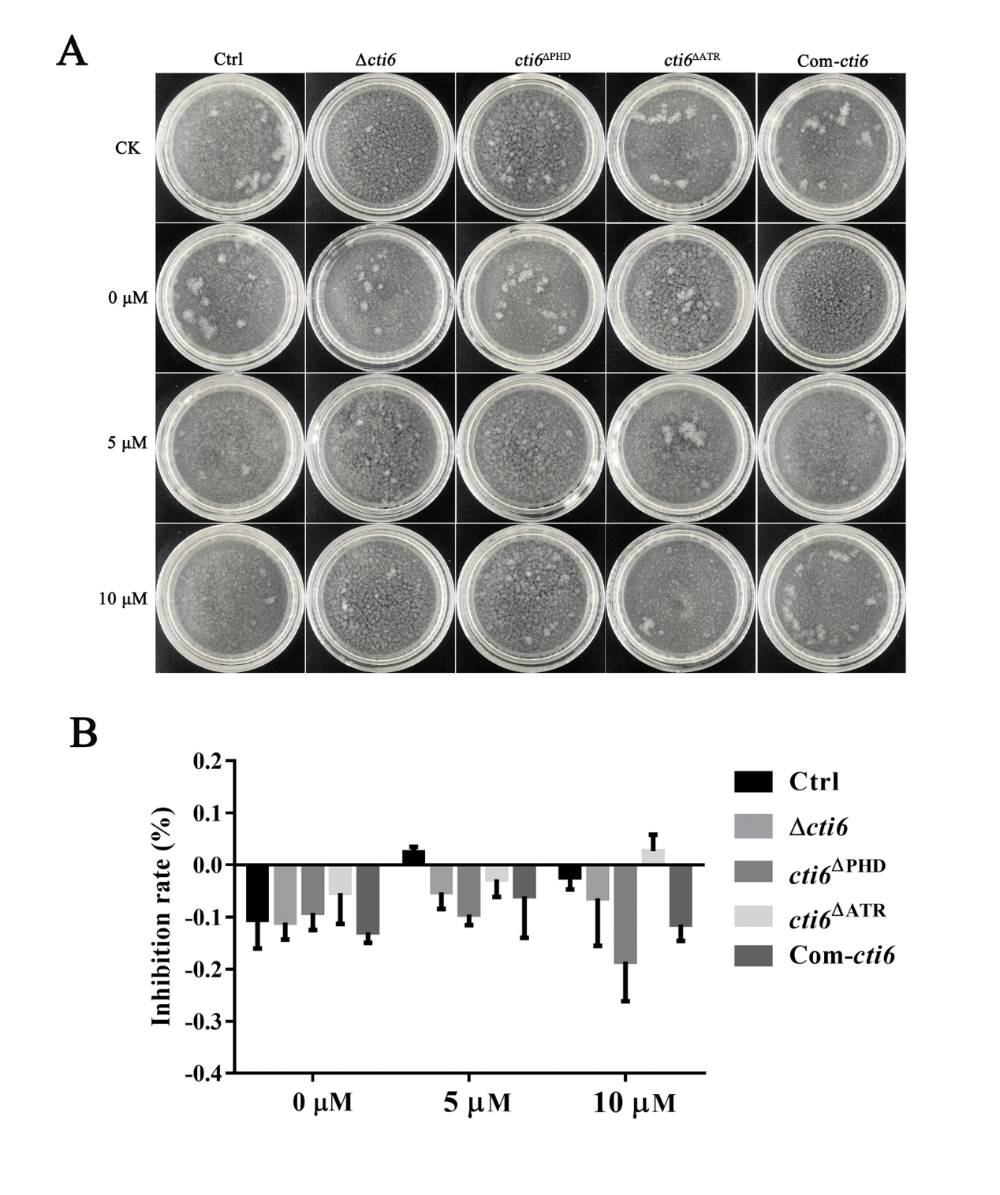


**Figure S3.** The iron stress effects in *A. flavus* strains. **A.** The *A. flavus* strains growth in different concentrations FeCl_3_. **B.** The dry weight were measured and represented with column graph according to the result of (A) panel


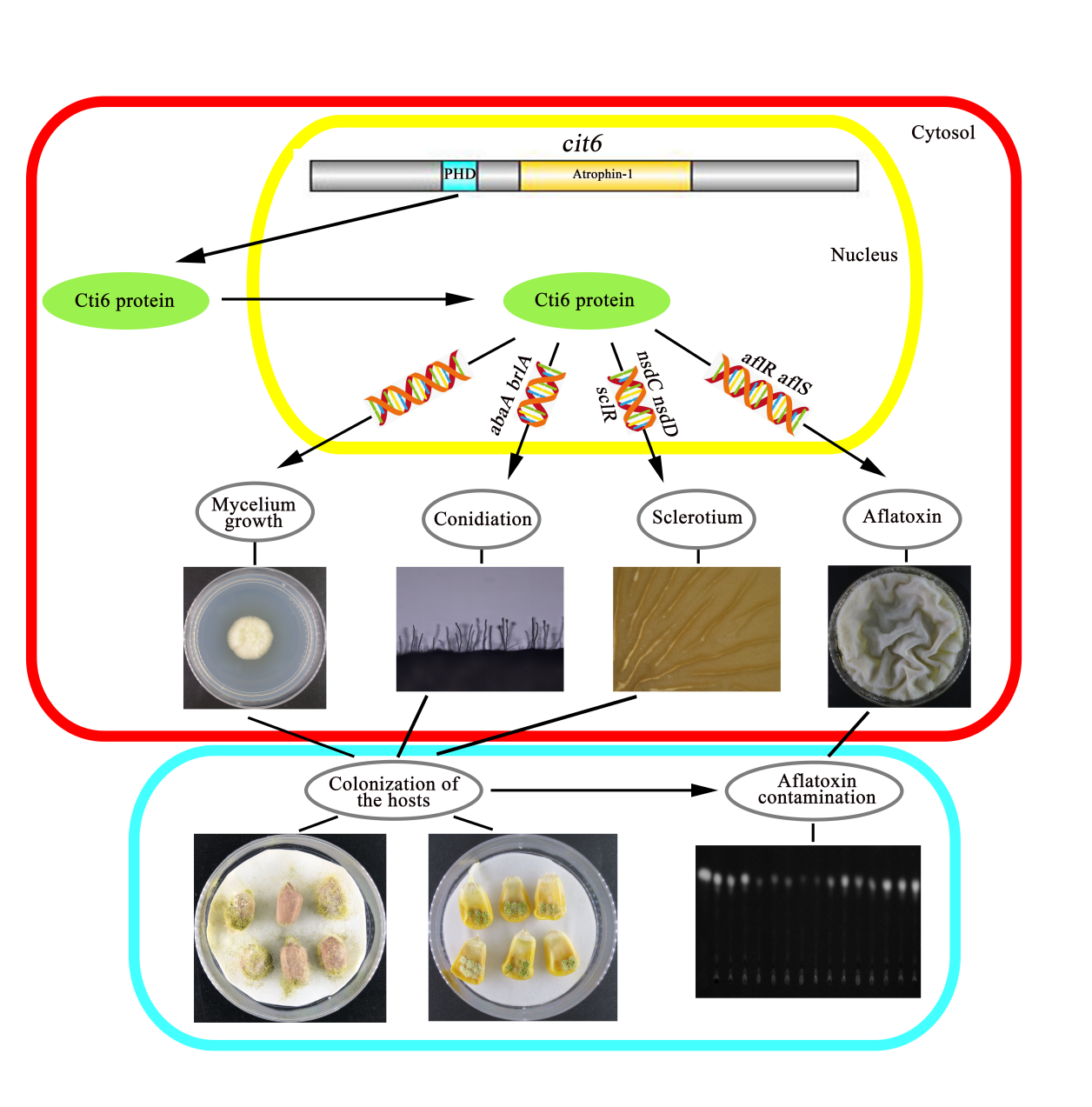


**Figure S4. The regulation model showing the pathways by which Cti6 regulates the morphogenesis and aflatoxin biological synthesis, and colonization of *A. flavus*.** Cti6 positively regulates hyphae growth of *A. flavus*, and dramatically boosts fungal sporulation through transcriptional factors BrlA and AbaA, initiates sclerotia formation by NsdC, NsdD and SclR, promotes AFB1 biological synthesis by AflR and AflS mediated orthodox aflatoxin pathway, thereby Cti6 plays a critical role in the colonization of the pathogenic fungus to crop grains by regulating hyphae growth, asexual reproduction and sclerotia formation.
